# The small molecule NSC676914A is cytotoxic and differentially affects NFκB signaling in ovarian cancer cells and HEK293 cells

**DOI:** 10.1186/s12935-014-0075-y

**Published:** 2014-08-12

**Authors:** Ethan Sagher, Lidia Hernandez, Callee Heywood, Gary T Pauly, Matthew R Young, Joel Schneider, Nancy H Colburn, Christina M Annunziata

**Affiliations:** 1 Women’s Malignancies Branch, Center for Cancer Research, National Cancer Institute, Bethesda 20892, MD, USA; 2 Chemical Biology Laboratory, Center for Cancer Research, National Cancer Institute, Frederick 21702, MD, USA; 3 Laboratory of Cancer Prevention, Center for Cancer Research, National Cancer Institute, Frederick 21702, MD, USA

**Keywords:** Ovarian cancer, NF-κB, IKKβ, NSC676914, Chemotherapy

## Abstract

**Background:**

The small molecule NSC676914A was previously identified as an NF-κB inhibitor in TPA-stimulated HEK293 cells (Mol Can Ther 8:571-581, 2009). We hypothesized that this effect would also be seen in ovarian cancer cells, and serve as its mechanism of cytotoxicity. OVCAR3 and HEK293 cell lines stably containing a NF-κB luciferase reporter gene were generated.

**Methods:**

Levels of NF-κB activity were assessed by luciferase reporter assays, after stimulation with LPA, LPS, TPA, and TNFα, in the presence or absence of a known NF-κB inhibitor or NSC676914A, and cytotoxicity was measured.

**Results:**

NSC676914A was toxic to both OVCAR3 and HEK293 cells. We also investigated the cytotoxicity of NSC676914A on a panel of lymphoma cell lines with well characterized mutations previously shown to determine sensitivity or resistance to NF-κB inhibition. The compound did not show predicted patterns of effects on NF-κB activity in either lymphoma, ovarian or HEK293 cell lines. In HEK293 cells, the small molecule inhibited NF-κB when cells were stimulated, while in OVCAR3 cells it only partially inhibited NF-κB. Interestingly, we observed rescue of cell death with ROS inhibition.

**Conclusions:**

The current study suggests that the effect of NSC676914A on NF-κB depends on cell type and the manner in which the pathway is stimulated. Furthermore, as it is similarly toxic to lymphoma, OVCAR3 and HEK293 cells, NSC676914A shows promising NF-κB-independent anti-cancer activity in ovarian tumor cells.

## Background

Ovarian cancer is frequently diagnosed in the late stages of the disease, and is the most common cause of death among gynecological cancers in women in the United States. Moreover, even as it only accounts for 3% of cancer cases in women, it is the fifth most common cause of death from all cancers [1]. The NF-κB family of gene transcription factors plays an important role in cell survival and proliferation, and constitutive NF-κB signaling has been identified in tumors of epithelial origin. Recent evidence suggests that this pathway also plays a role in ovarian cancer; NF-κB activation has been shown to increase the aggressiveness of ovarian cancer cell lines [2], and overexpression of the NF-κB subunit p50 has been shown to be positively correlated with poor outcome among ovarian cancer patients [3]. NF-κB signaling is therefore a potential target for therapeutic treatment of this disease.

Platinum-based and taxane-based chemotherapy are staples in the treatment of ovarian cancer. Even so, the relapse rates for ovarian cancer patients are extremely high [4], which emphasizes the importance of exploring new therapeutic agents. NSC676914 was recently identified as an NF-κB inhibitor in a high-throughput screen of a synthetic library aimed at identifying AP-1 inhibitors [5], and shown to inhibit NF-κB transcriptional activity at low concentrations in TPA-stimulated HEK293 cells. That previous study tested a mixture of compounds. For the work we present in this manuscript, we purified an active component, here designated NSC676914A, and determined the structure (Additional file 1: Figure S1A). The material used in this study is newly synthesized pure NSC676914A. In this study we hypothesized that this small molecule could be selectively toxic to ovarian cancer cells that rely on NF-κB signaling for proliferation and survival. We discovered, however, a broader applicability of this compound across cancers, with reasonable activity against ovarian cancer cell lines.

## Results

**Figure 1:**
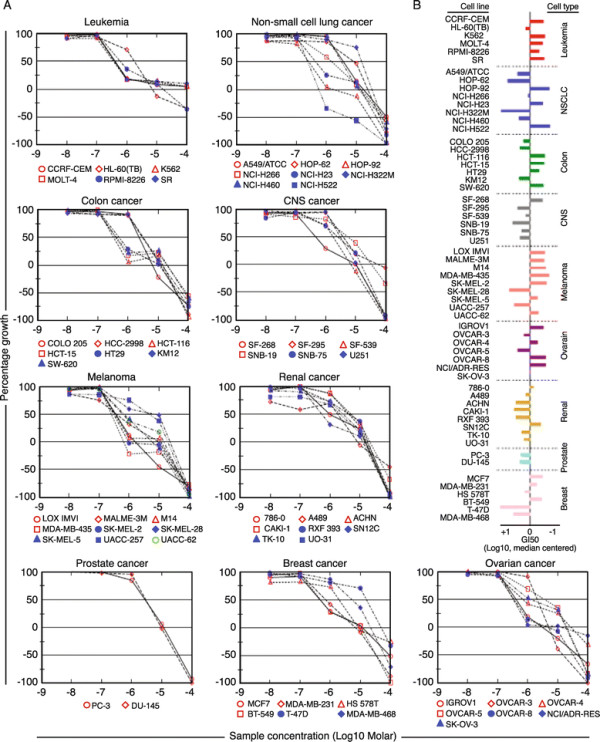
**NSC676914A shows differential toxicity to NCI-60 cancer lines. (A)** Growth inhibition of NCI-60 cancer cell lines after exposure to NSC676914A. NCI-60 cancer panel cells are plated for 24 h prior to addition of compound. Cells are then incubated for an additional 48 h and cell number estimated by Sulforhodamine B staining as described. **(B)** GI50 is the calculated micromolar concentration resulting in 50% reduction in the measured protein at the end of drug treatment compared to that at the beginning. The median log GI50 for NSC676914A over all cell lines was −5.91.

In a previous study [4] using HEK293 cells, NSC676914A was shown to inhibit NF-κB activity in vitro at low micromolar concentrations in a dose-dependent manner. A purified version of the compound was recently synthesized, and submitted to the NCI-60 tumor cell panel for growth inhibition analysis (Figure 1A). Results showed an overall tumor cell median GI50 of −5.91, with greater sensitivities found in the leukemia, melanoma, colon and ovarian cancer cell groups (Figure 1B, Additional file 2: Table S1). Within the ovarian cancer cell panel, NSC676914A caused 50% or more growth inhibition of 7 ovarian cell lines at concentrations between 1 and 10 μM, the same concentration at which NF-κB was inhibited in HEK293 cells [5].

BAY-7085 (NSC663627) is a known inhibitor of NF-κB, and we sought to determine whether its pattern of toxicity resembled that of NSC676914A. This compound resulted in only mild toxicity to the ovarian cancer cell lines in the NCI-60 panel (Additional file 3: Figure S2A). A COMPARE analysis of BAY-7085 indentified a number of highly correlated compounds of known NF-κB inhibitory activity (Additional file 3: Figure S2A, B). This analysis suggested a wide variation in patterns of toxicity of compounds inhibiting NF-κB activity.

**Figure 2:**
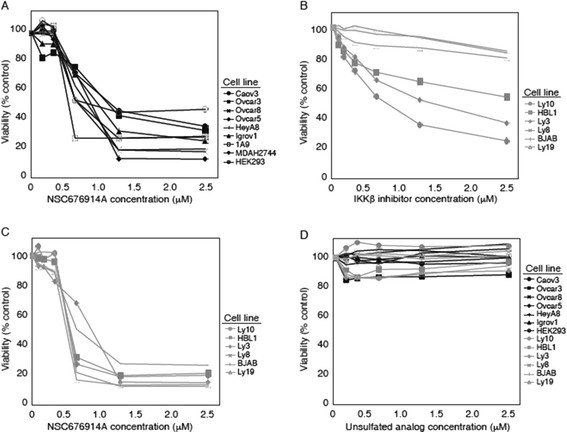
**NSC676914A inhibits HEK293 and ovarian cancer cell proliferation. (A)** Proliferation of HEK293 and a panel of ovarian cancer cell lines after 72 h treatment with varying concentrations of NSC676914A. Cell viability was assessed by XTT assay as described and reported as percent untreated control. Data are representative of 3 experiments. **(B)** Viability of 3 human lymphoma lines (open markers) sensitive to NFκB inhibition, and 3 resistant lymphoma lines (closed markers) after 72 h treatment with IKKβ inhibitor. **(C)** Viability of ovarian cancer cell lines after 72 h exposure to NSC676914A. **(D)** Proliferation of the same panel of ovarian lines in **(A)**, plus above described lymphoma lines after treatment with the inactive NSC676914A unsulfated alcohol analog.

In order to clarify the mechanism of NSC676914A toxicity in ovarian cancer cells, we assessed the sensitivity of an expanded ovarian cancer cell panel and HEK293 cells to the compound at low micromolar concentrations. NSC676914A caused 50% growth inhibition for all the cell lines between the range of 0.5-1.25 μM. We also investigated the effect of NSC676914A treatment on a panel of lymphoma cell lines with well characterized mutations previously shown to determine sensitivity or resistance to NF-κB inhibition [6]. This panel of cell lines could demonstrate the point at which a compound interferes with NF-κB signaling, based on the pattern of sensitivity. The IKKβ -dependent cell lines carry a mutation upstream of IKKβ, that activates the kinase. The IKKβ -independent lines do not depend on IKKβ activity for survival. As shown in Figure 2B, upon treatment with IKKβ inhibitor, sensitive lines presented significant growth inhibition at 0.5-2 micromolar concentrations, while resistant lines required at least 5–10 times the dose. NSC676914A was equally toxic to all lymphoma cell lines (Figure 2C), while its unsulfated alcohol analog was non-toxic to all ovarian and lymphoma cell lines (Figure 2D). The unsulfated alcohol analog (Additional file 1: Figure S1B) was included as a control since previous studies showed it to be inactive in inhibiting NF-κB in HEK293 cells. Taken together, these data suggest that NSC676914A is toxic to IKKβ-dependent and -independent lymphoma lines, and therefore must act by a different mechanism.

**Figure 3:**
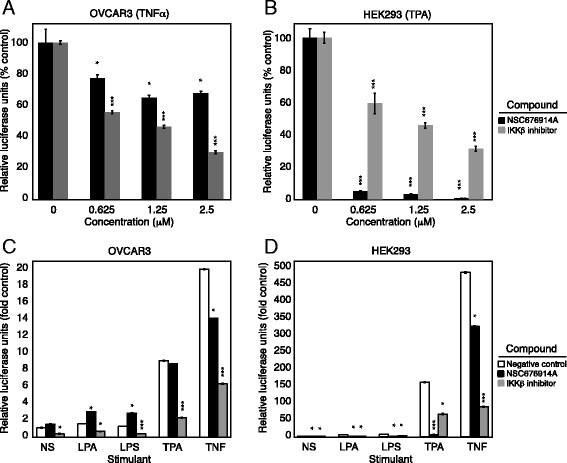
**NSC676914A differentially affects NF-κB transcriptional activity. (A)** NF-κB activity following 18 h treatment with varying concentrations of NSC676914 and IKKβ inhibitor, during stimulation of OVCAR3 cells with TNFα **(A)** and HEK293 cells with TPA **(B)**, as described. NF-κB activity following 18 h treatment with varying concentrations of NSC676914A or IKKβ inhibitor, during stimulation of OVCAR3 cells **(C)** and HEK293 cells **(D)** with 1 μM LPA, 1 μg/ml LPS, 10nM TPA and 10 ng/ml TNFα as described. Significant differences in treated OVCAR3 activity were compared between cells with each stimulant and no inhibitors present, as indicated. * p < 0.01, **p < 0.001, ***p < 0.0001.

We next sought to investigate the effect of NSC676914A on NF-κB signaling in HEK293 cells. We measured NF-κB transcriptional activity of HEK293 cells transiently transfected with a luciferase reporter plasmid. Indeed, the compound significantly inhibited NF-κB activity (72% and 61% inhibition at 10 μM and 2 μM concentrations, respectively) in TPA-stimulated cells after 18 h treatment, confirming earlier findings (Additional file 4: Figure S3A). The published studies were performed with the unpurified compound, and using transiently transfected HEK293 cells. In order to compare HEK293 cells and ovarian cancer cells, we established stable OVCAR3 and HEK293 cell lines expressing a luciferase reporter gene responding to an NF-κB transcriptional regulatory element. Under these new conditions, the pure NSC676914A partially blocked NF-κB activity induced by TNFα stimulation for 18 h in OVCAR3, but was more effective in HEK293 cells under stimulation with TPA (Figure 3A, B). The higher concentrations of the impure compound were previously shown to cause growth inhibition after 72 h exposure. In our current study, IKKβ inhibition caused a 40-70% dose-dependent decrease in HEK293 luciferase activity, while pure NSC676914A produced a dose-dependent 95-98% decrease. OVCAR3 cells, in turn, showed a 45-70% decrease in luciferase activity after IKKβ inhibition, however NSC676914A produced only a 23-33% decrease, suggesting that this compound is a much more potent NF-κB activity inhibitor in TPA-stimulated HEK293 cells than in TNFα-stimulated OVCAR3 cells.

Ovarian cancer cells respond to a number of different physiological stimuli. We therefore investigated the NF-κB inhibition achieved by NSC676914A in both OVCAR3 and HEK293 reporter lines, following stimulation by LPA, LPS, TPA or TNFα in order to clarify whether the compound had a greater effect in a particular pathway triggering NF-κB activation. We chose to use the concentration of 1.25 μM, which achieves the maximum level of NF-κB inhibition under stimulation with TNFα, and beyond which there is no further inhibition (Figure 3A). NSC676914A did not inhibit constitutive NF-κB signaling in OVCAR3 cells; HEK293 cells had very little constitutive NF-κB activity (Additional file 4: Figure S3B). OVCAR3 cells responded minimally to LPA and LPS stimulation, while TPA and TNFα caused the greatest increase in NF-κB activation (Figure 3C); IKKβ inhibition decreased luciferase activity in all cases by 50-70%, while NSC676914A had minimal (4-30%) effects. Similarly, in HEK293 cells, TNFα and TPA had the greatest ability to activate NF-κB (Figure 3D). In these cells, however, NSC676914A was more effective than IKKβ inhibitor in blocking NF-κB activity after TPA stimulation (98% decrease). These data suggest that NSC676914A has a cell-specific and pathway-specific mechanism of inhibiting NF-κB activity. It is unclear, however, to what extent the inhibition of NF-κB is the reason for the cytotoxicity seen.

**Figure 4:**
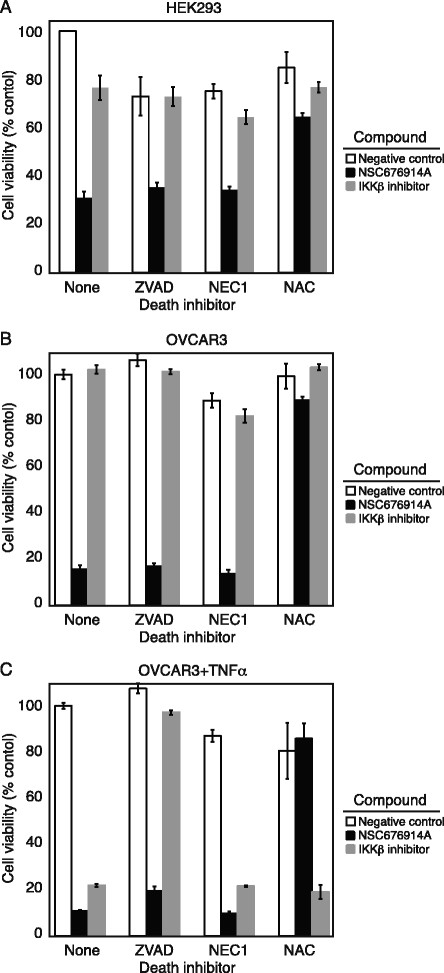
**NSC676914A cytotoxicity is rescued by ROS inhibitor.** Cell death in parental HEK293 and OVCAR3 cells after 3 days of exposure to NSC676914A or IKKβ inhibitor. Viability of HEK293 cells **(A)** or OVCAR3 cells **(B)** and TNFα-treated OVCAR3 **(C)** cells, after 72 h exposure to NSC676914A or IKKβ inhibitor, following 1 h pretreatment with cell death inhibitors ZVAD 10 μM, NEC-1 20 μM, NAC 1 mM, as described in Methods. Data are average of three independent measurements.

In order to clarify whether the mechanism by which NSC676914A is toxic in HEK293 cells is also responsible for killing cancer cells, parental HEK293 and OVCAR3 cells were pre-treated with 3 specific inhibitors: Caspase-mediated death (ZVAD), caspase-independent necroptosis (NEC-1), and ROS-mediated death (NAC), prior to treatment with NSC676914A or IKKβ inhibitor. OVCAR3 cells were treated in the presence or absence of TNFα. NSC676914A is more toxic to unstimulated HEK293 and OVCAR3 cells than IKKβ inhibition (Figure 4A, B). The ROS-inhibitor NAC completely rescued cell death induced by NSC676914A, but ZVAD or NEC-1 were ineffective, suggesting that cell death in unstimulated cells does not proceed through an apoptotic or necroptotic mechanism. However, TNFα-stimulated OVCAR3 cells are killed by both NSC676914A and IKKβ inhibition (Figure 4C). The cell death that follows TNFα and IKKβ inhibition clearly involves a caspase-dependent cell death that can be rescued by ZVAD, while NSC676914A only induces a ROS-dependent cell death. ROS production by NSC676914A was confirmed (Additional file 5: Figure S4).

## Discussion

Our data suggest that NF-κB activity in HEK293 cells is inhibited by NSC676914A at low concentrations; however this effect is not its primary mechanism of toxicity, as IKKβ inhibition alone is less toxic to the cells. In addition, in a well characterized panel of lymphoma cell lines, NSC676914A was equally toxic to cell lines known to be dependent or independent of IKKβ signaling, consistent with the idea that NSC676914A kills cells by a mechanism other than IKKβ inhibition. Further, in TNFα-stimulated ovarian cancer cells this compound was equally toxic as IKKβ inhibition; however the IKKβ inhibitor clearly proceeded through a caspase-dependent mechanism while NSC676914A did not. These findings suggest that the NF-κB inhibitory activity of NSC676914A is specific to the signaling pathway triggered by TPA stimulation in HEK293 cells, which is not the mechanism by which it kills cells.

The NCI-60 panel of cell lines shows that NSC676914A has some preference for ovarian cancer cell lines, among a few other cell types. While its exact target remains unknown, it displays broad toxicity to 10 ovarian cancer cell lines, in our hands and the NCI-60 panel. This includes the drug-resistant cell line, NCI/ADR-RES in the NCI-60 panel. There was the least amount of cell killing in the lung cancer, kidney cancer and CNS cancer cell lines in the NCI-60, suggesting that the drug is not broadly toxic to growing cell lines, but displays some degree of specificity to ovarian cancer. Interestingly, an earlier unpurified version of NSC676914 was able to potentiate topotecan killing of neuroblastoma cells [7].

Future work with the NSC676914A compound could include investigation into its specific targets within the cell, its differential effects on NF-κB signaling in HEK293 cells as opposed to OVCVAR3 cells, and its mechanism of inducing cell death overall. We found that its cytotoxic activity was not blocked by inhibitors of apoptosis or necroptosis, but it was deactivated by pretreatment with NAC, a scavenger of ROS. Many anti-cancer agents increase reactive oxygen species as a means to kill cancer cells, thus making this a reasonable therapeutic strategy [8].

## Conclusions

NSC676914A shows promising anti-cancer activity in a range of cell lines, including ovarian cancer, regardless of their dependence on NF-κB signaling. Future studies should further investigate its cellular target in order to optimize its delivery in the clinical setting.

## Methods

### Reagents

NSC676914A (Additional file 1: Figure S1A) was synthesized by diazotization of 2-[(4-aminophenyl)sulfonyl]-1-hydrogen sulfate followed by coupling with m-toluidine. An unsulfated alcohol analog (Additional file 1: Figure S1B) was similarly prepared from 2-[(4-aminophenyl)sulfonyl]-1-ethanol. Both compounds were purified to >95% purity by reverse phase HPLC. DMSO stocks were used for all experiments. IKKβ inhibitor (IKK-2 Inhibitor IV [5-(*p*-Fluorophenyl)-2-ureido]thiophene-3-carboxamide, catalog #401484) (Additional file 1: Figure S1C) was purchased from EMD Biosciences (La Jolla, CA). LPA (857130C) was purchased from Avanti Polar Lipids (Alabaster, AL). LPA stocks were made in PBS containing 1% fatty acid-free bovine serum albumin. TPA (4174) was purchased from Cell Signaling Technology, Inc (Danvers, MA). Recombinant Human TNFα (300-01A) was purchased from Peprotech (Rocky Hill, NJ). Z-VAD-FMK (2163) and Necrostatin-1 (2324) were purchased from Tocris Bioscience (Ellisville, MO). Puromycin, XTT, PMS, LPS (L5293) and N-Acetyl-L-cysteine (A7250) were purchased from Sigma-Aldrich (St. Louis, MO).

### Cell lines and culture conditions

Ovarian cancer cell line OVCAR3 was a gift from Elise Kohn (NCI, Bethesda, MD), HEYA8 was a gift from Gordon Mills (MD Anderson Cancer Center, Houston, TX), and 1A9 cells were a gift from A.T. Fojo (NCI, Bethesda, MD); OVCAR5, OVCAR8, and IGROV1 cells were from the NCI-Frederick Developmental Therapeutics Program tumor/cell line repository (Frederick, MD). Human embryonic kidney (HEK293) MDAH2744 and CAOV3 cells were obtained from ATCC. Lymphoma cell lines Ly10, HBL1, Ly3, Ly8, BJAB and Ly8 were a gift from L. Staudt (NCI, Bethesda, MD). Ovarian and lymphoma cancer cell lines were cultured in RPMI 1640, HEK293 was cultured in DMEM, all containing 10% fetal bovine serum (Hyclone, Pittsburgh, PA) and standard antibiotics. All media were obtained from Invitrogen (Carlsbad, CA). Cell lines were authenticated in July 2009 at the Johns Hopkins University Fragment Analysis Facility (Baltimore, MD), using Promega PowerPlex 1.2 System to test for 8 STR markers (D16S539, D7S820, D13S317, D5S818, CSF1PO, TPOX, THO1, vWA) and amelogenin for gender determination. Authenticity was confirmed against the ATCC database www.atcc.org/CulturesandProducts/CellBiology/STRProfileDatabase/tabid/174/Default.aspx) CLIMA database (http://drcat.sourceforge.net/clima.html) and NCI-60 database published data [9].

### NCI-60 tumor cell panel

Cytotoxicity screen of NSC676914A was performed by the Developmental Therapeutics Program of NCI (NIH), as described (http://dtp.nci.nih.gov). The synthesized compound was submitted for testing in the NCI-60 panel on February 25, 2013. COMPARE analysis was performed through the DTP website as described [10].

### Luciferase reporter assays

HEK293 cells transiently transfected as described with an NF-κB reporter [5] were treated with NSC676914A or an alcohol analog lacking the sulfate group, previously shown to be inactive in HEK293 cells. After TPA treatment, NF-κB activity was measured using the Luciferase Assay System as per manufacturer’s instructions. OVCAR3 and HEK293 cell lines were transduced with a lentiviral construct containing an NF-κB reporter gene (CLS-013 L), which was obtained from SABiosciences (Frederick, MD), according to the manufacturer’s specifications. At the conclusion of the transduction, successfully transduced cells were selected using 1 μg/ml puromycin for two weeks, and stable cell lines were maintained. The Luciferase Assay System (E1501) was purchased from Promega (Madison, WI) to measure NFκB activity, which was done following the manufacturer’s protocol. In brief, cells were seeded at a density of 10,000 cells/well in 96-well plates for 24 hours, and then serum-starved with 0.5% FBS for 24 hours. From concentrated stocks, NFκB inhibitors (0–2.5 μM) were added, followed by varying concentrations of stimulants LPA (1 μM), TPA (10 nM), LPS (1 μg/ml) and TNFα (10 ng/ml) one hour later. After 18 hours of incubation, cells were washed with PBS, lysed and Luciferase Assay Reagent (LAR) added. Luminescence was measured in a Molecular Devices (Sunnyvale, CA) SpectraMax M5 Multi-Mode Microplate Reader using the Dual-Luciferase_SpectraMax L protocol. Data were normalized to cell number measured by XTT cell viability assay [11] in duplicate plates.

### Cell viability assays

Attached cell growth was assessed using XTT as described [11]. Cells were seeded in 96-well plates at a density of 2,000-5,000 cells/100 μl/well and incubated for up to 72 hours after NSC676914A (0–2.5 μM) was added. In experiments using cell death inhibitors, CellTiter-Glo Luminescent Cell Viability Assay (G7571) was purchased from Promega (Madison, WI) and performed following the manufacturer’s protocol. In brief, cells were pretreated with the pan-caspase inhibitor Z-VAD-FMK (ZVAD, 10 μM), necroptosis inhibitor Necrostatin 1 (NEC-1, 20 μM), or ROS inhibitor N-Acetyl-L-cysteine (NAC, 1 mM) for one hour. In OVCAR3 cells medium was supplemented with 10 ug/ml TNFα. NSC676914A or IKKβ Inhibitor was then added at 1.25 μM, and all cultures incubated for 72 hours before measuring luminescence with a Molecular Devices SpectraMax M5 Multi-Mode Microplate Reader, using the CellTiter-Glo_SpectraMax L protocol.

### Statistical analyses

Experiments were conducted using 6 replicates of each experimental condition. Results were analyzed for significant differences using two-tailed T-tests in Microsoft Excel.

### Reactive oxygen species detection assays

Please see Additional file 6.

## Abbreviations

IKK: Inhibitor of IκB kinase

LPA: Lysophosphatidic acid

LPS: Lipopolysaccharide

NAC: N-Acetyl-L-cysteine

NEC: Necrostatin

NF-κB: Nuclear factor kappa B

ROS: Reactive oxygen species

TNF: Tumor necrosis factor

TPA: Tetradecanoyl phorbol acetate

XTT: 2,3-Bis-(2-Methoxy-4-Nitro-5-Sulfophenyl)-2H-Tetrazolium-5-Carboxanilide

ZVAD: Z-Val-Ala-DL-Asp-fluoromethylketone

## Competing interests

The authors declare no competing interests.

## Authors’ contributions

CH performed transient cell line reporter assays, ES derived stable reporter cell lines, performed reporter assays, cell death assays and data analysis. LH participated in the derivation of cell lines, reporter assays and cytotoxicity assays of lymphoma and ovarian cell lines and drafted the manuscript. GTP synthesized, purified and analyzed the structure of NSC676914A and the unsulfated alcohol analog. MRY performed transient reporter assays and analyzed data. JS and NHC contributed to the conception and design of studies and manuscript revisions. CMA contributed to study conception and design, data interpretation, manuscript preparation and revisions. All authors read and approved the final manuscript.

## Additional files

## Supplementary Material

Additional file 1: Figure S1. Chemical structures of compounds used in the study. (A) NSC676914A, (B) the unsulfated alcohol analog, (C) the commercially obtained specific IKKβ inhibitor [5-(*p*-Fluorophenyl)-2-ureido]thiophene-3-carboxamide. Click here for file

Additional file 2: Table S1. GI50 values for NSC676914A cytotoxicity in NCI-60 cell panel. Click here for file

Additional file 3: Figure S2. NCI-60 cell growth inhibition pattern of NF-κB inhibitors. (A) Growth inhibition of NCI-60 cancer cell lines after exposure to NF-κB inhibitor BAY 11-7085 and others. NCI-60 cancer panel cells are plated for 24 h prior to addition of compound. Cells are then incubated for an additional 48 h and cell number estimated by Sulforhodamine B staining as described. (B) COMPARE analysis of toxicity correlations between other inhibitors and BAY 11-7085 performed through DTP website as described. Click here for file

Additional file 4: Figure S3. NF-κB reporter activity with analogs of NSC676914A. (A) HEK 293 cells were transiently transfected with an NF-κB luciferase reporter construct and helper constructs as described in Methods. Cells were pretreated with the indicated concentrations of compounds for 1hour and stimulated with 10 nM TPA for 18 h; luciferase reporter activity was measured as described, and calculated as percent of control. (B) NF-κB signaling in OVCAR3 and HEK293 cells stably expressing reporter vector under no stimulation, as described in Methods. NSC676914A had no effect on constitutive NF-κB activity. Click here for file

Additional file 5: Figure S4. Reactive Oxygen Species (ROS) Levels in OVCAR3 cells after treatment with NSC676914A. DCFDA levels measured after 2 hours after treatment of OVCAR3 cells with known inducer of ROS 400 μM H2O2 (positive control), and 1.25 μM NSC676914A, as described in Additional file 6. NSC676914A produces an increase in ROS in OVCAR3 cells. Click here for file

Additional file 6: Reactive oxygen species detection assays. Click here for file
