# Supplementary figures and images for: The small molecule NSC676914A is cytotoxic and differentially affects NFκB signaling in ovarian cancer cells and HEK293 cells
Source: Cancer Cell Int. 2014 Aug 12;14:75. doi: 10.1186/s12935-014-0075-y (PMC4198909; doi:10.1186/s12935-014-0075-y)

A

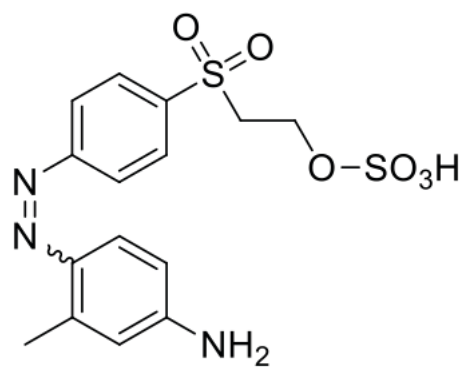

B

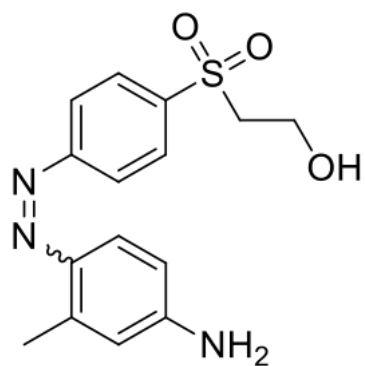

C

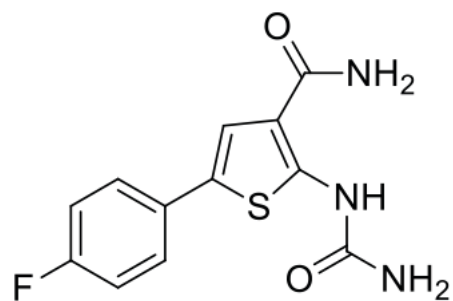

Supplement: Additional file 1: Figure S1. — Chemical structures of compounds used in the study. (A) NSC676914A, (B) the unsulfated alcohol analog, (C) the commercially obtained specific IKKβ inhibitor [5-(p-Fluorophenyl)-2-ureido]thiophene-3-carboxamide. [file s12935-014-0075-y-S1.pdf]

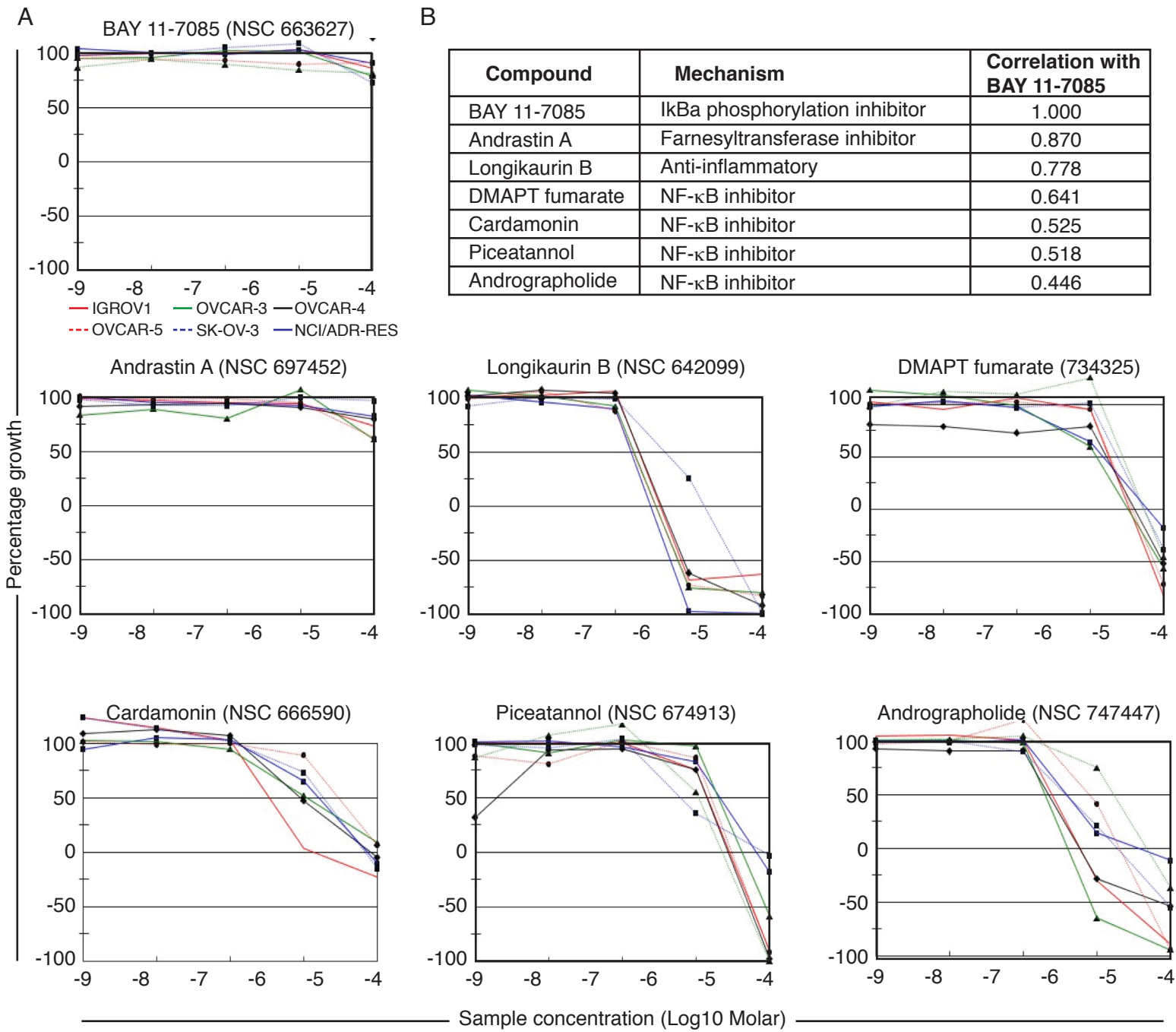

Supplement: Additional file 3: Figure S2. — NCI-60 cell growth inhibition pattern of NF-κB inhibitors. (A) Growth inhibition of NCI-60 cancer cell lines after exposure to NF-κB inhibitor BAY 11-7085 and others. NCI-60 cancer panel cells are plated for 24 h prior to addition of compound. Cells are then incubated for an additional 48 h and cell number estimated by Sulforhodamine B staining as described. (B) COMPARE analysis of toxicity correlations between other inhibitors and BAY 11-7085 performed through DTP website as described. [file s12935-014-0075-y-S3.pdf]

A

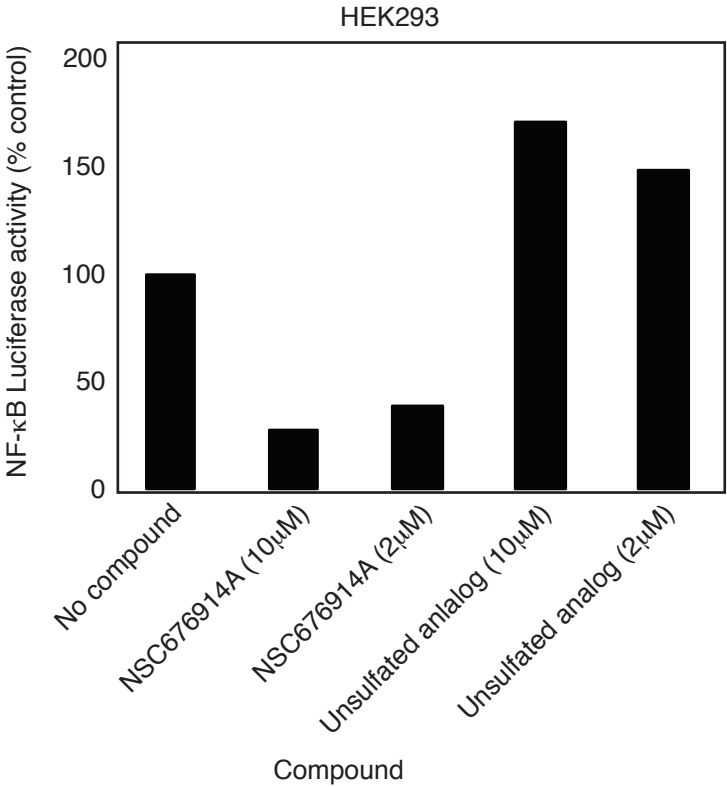

B

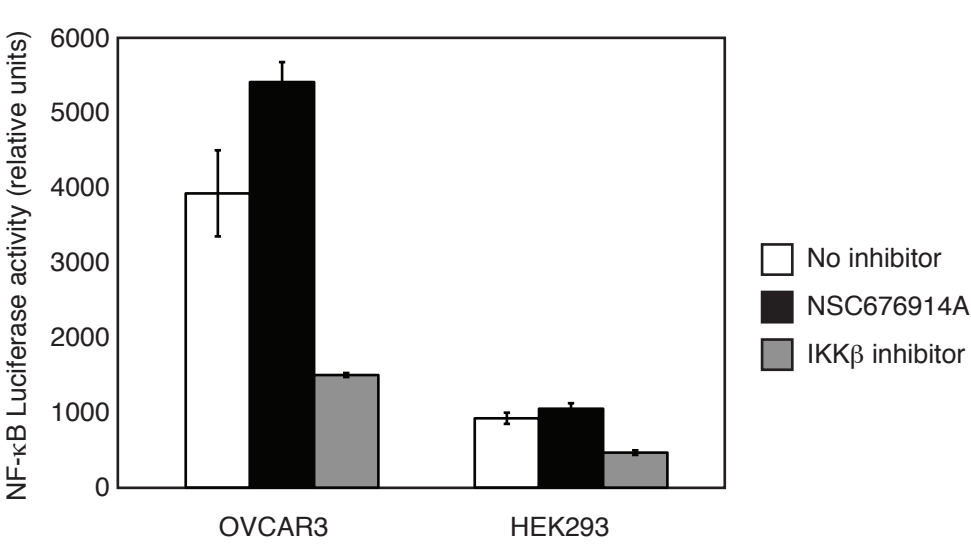

Supplement: Additional file 4: Figure S3. — NF-κB reporter activity with analogs of NSC676914A. (A) HEK 293 cells were transiently transfected with an NF-κB luciferase reporter construct and helper constructs as described in Methods. Cells were pretreated with the indicated concentrations of compounds for 1hour and stimulated with 10 nM TPA for 18 h; luciferase reporter activity was measured as described, and calculated as percent of control. (B) NF-κB signaling in OVCAR3 and HEK293 cells stably expressing reporter vector under no stimulation, as described in Methods. NSC676914A had no effect on constitutive NF-κB activity. [file s12935-014-0075-y-S4.pdf]

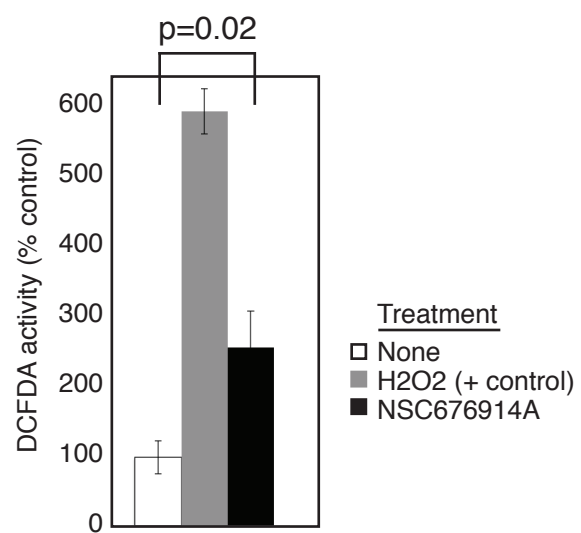

Supplement: Additional file 5: Figure S4. — Reactive Oxygen Species (ROS) Levels in OVCAR3 cells after treatment with NSC676914A. DCFDA levels measured after 2 hours after treatment of OVCAR3 cells with known inducer of ROS 400 μM H2O2 (positive control), and 1.25 μM NSC676914A, as described in Additional file 6. NSC676914A produces an increase in ROS in OVCAR3 cells. [file s12935-014-0075-y-S5.pdf]
