# Supplementary material for: The small molecule NSC676914A is cytotoxic and differentially affects NFκB signaling in ovarian cancer cells and HEK293 cells
Source: Cancer Cell Int. 2014 Aug 12;14:75. doi: 10.1186/s12935-014-0075-y (PMC4198909; doi:10.1186/s12935-014-0075-y)
Supplement: Additional file 2: Table S1. — GI50 values for NSC676914A cytotoxicity in NCI-60 cell panel. [file s12935-014-0075-y-S2.pdf]

Supplementary Table 1: GI50 values for NSC676914A in NCI60 cell line screen

Cell type	Cell line	GI50 (log10 Molar)	GI50 (Molar)	GI50 (microM)
Leukemia	CCRF-CEM	-6.40	3.98107E-07	0.40
	HL-60(TB)	-5.75	1.77828E-06	1.78
	K-562	-6.45	3.54813E-07	0.35
	MOLT-4	-6.36	4.36516E-07	0.44
	RPMI-8226	-6.22	6.0256E-07	0.60
	SR	-6.41	3.89045E-07	0.39
NSCLC	A549/ATCC	-5.43	3.71535E-06	3.72
	HOP-62	-5.07	8.51138E-06	8.51
	HOP-92	-6.59	2.5704E-07	0.26
	NCI-H266	-5.82	1.51356E-06	1.51
	NCI-H23	-6.36	4.36516E-07	0.44
	NCI-H322M	-4.82	1.51356E-05	15.14
	NCI-H460	-5.49	3.23594E-06	3.24
	NCI-522	-6.62	2.39883E-07	0.24
Colon	COLO205	-5.63	2.34423E-06	2.34
	HCC-2998	-5.52	3.01995E-06	3.02
	HCT-116	-6.45	3.54813E-07	0.35
	HCT-15	-6.40	3.98107E-07	0.40
	HT29	-6.24	5.7544E-07	0.58
	KM12	-5.50	3.16228E-06	3.16
	SW-620	-6.38	4.16869E-07	0.42
CNS	SF-268	-6.33	4.67735E-07	0.47
	SF-295	-5.42	3.80189E-06	3.80
	SF-239	-5.75	1.77828E-06	1.78
	SNB-18	-5.25	5.62341E-06	5.62
	SNB-75	-5.59	2.5704E-06	2.57
	U251	-5.51	3.0903E-06	3.09
Melanoma	LOXIMVI	-6.44	3.63078E-07	0.36
	MALME-3M	-6.40	3.98107E-07	0.40
	M14	-6.47	3.38844E-07	0.34
	MDA-MB-435	-6.59	2.5704E-07	0.26
	SK-MEL-2	-6.51	3.0903E-07	0.31
	SK-MEL-28	-5.11	7.76247E-06	7.76
	SK-MEL-5	-6.16	6.91831E-07	0.69
	UACC-257	-5.32	4.7863E-06	4.79
	UACC-62	-6.20	6.30957E-07	0.63
Ovarian	IGROV1	-6.39	4.0738E-07	0.41
	OVCAR-3	-5.69	2.04174E-06	2.04
	OVCAR-4	-6.16	6.91831E-07	0.69
	OVCAR-5	-5.43	3.71535E-06	3.72
	OVCAR-8	-6.46	3.46737E-07	0.35
	NCI/ADR RES	-6.47	3.38844E-07	0.34
	SK-OV-3	-5.89	1.28825E-06	1.29
Renal	786-0	-6.01	9.77237E-07	0.98
	A489	-5.74	1.8197E-06	1.82
	ACHN	-5.37	4.2658E-06	4.27
	CAKI-1	-5.30	5.01187E-06	5.01
	RXF 393	-5.38	4.16869E-06	4.17
	SN 12C	-6.28	5.24807E-07	0.52
	TK-10	-5.57	2.69153E-06	2.69
	UO-31	-5.66	2.18776E-06	2.19
Prostate	PC-3	-5.56	2.75423E-06	2.75
	DU-145	-5.53	2.95121E-06	2.95
Breast	MCF7	-6.34	4.57088E-07	0.46
	MDA-MB-231	-6.15	7.07946E-07	0.71
	HS 578 T	-5.70	1.99526E-06	2.00
	BT-549	-6.31	4.89779E-07	0.49
	T-47D	-4.80	1.58489E-05	15.85
	MDA-MB-468	-5.28	5.24807E-06	5.25
