# Supplementary material for: The small molecule NSC676914A is cytotoxic and differentially affects NFκB signaling in ovarian cancer cells and HEK293 cells
Source: Cancer Cell Int. 2014 Aug 12;14:75. doi: 10.1186/s12935-014-0075-y (PMC4198909; doi:10.1186/s12935-014-0075-y)
Supplement: Additional file 6: — Reactive oxygen species detection assays. [file s12935-014-0075-y-S6.docx]

**SUPPLEMENTARY METHODS**

*Reactive Oxygen Species Detection Assays*

OVCAR3 cells were plated in opaque 96-well plates at a density of 40,000 cells/well. After 24 hours, wells were washed with PBS and cells treated with 2.5µM NSC676914A or 400µM H2O2 as a positive control. After 2 h , 2.5µM CM-H2DCFDA (Invitrogen, cat# C6827) from a DMSO stock was added according to manufacturer’s specifications, and plates were incubated in the dark for 40 minutes. Fluorescence was measured using a Molecular Devices SpectraMax M5 microplate reader at an excitation wavelength of 485nm and emission of 530nm,
